# Overlooked by the obstetric gaze – how women with persistent health problems due to severe perineal trauma experience encounters with healthcare services: a qualitative study

**DOI:** 10.1186/s12913-024-11037-5

**Published:** 2024-05-09

**Authors:** Katharina Tjernström, Inger Lindberg, Maria Wiklund, Margareta Persson

**Affiliations:** 1 Department of Nursing, Umeå University, 901 87 Umeå, Sweden; 2 Department of Community Medicine and Rehabilitation, Section of Physiotherapy, Umeå University, 901 87 Umeå, Sweden

**Keywords:** Severe perineal trauma, Persistent health problems, Qualitative content analysis, Healthcare encounters, Postpartum healthcare, Normalisation, Key person, Access to care, Empowerment

## Abstract

**Background:**

During the first year postpartum, about 25 per cent of Swedish women with severe perineal trauma (SPT), i.e., a third- or fourth-degree perineal laceration at childbirth, are unsatisfied with their healthcare contacts. Further, there is a lack of research on the more long-term experiences of healthcare encounters among women with persistent SPT-related health problems. This study explores how women with self-reported persistent SPT-related health problems experience their contact with healthcare services 18 months to five years after childbirth when the SPT occurred.

**Methods:**

In this descriptive qualitative study, a purposive sample of twelve women with self-reported persistent health problems after SPT were individually interviewed from November 2020 – February 2022. The data was analysed using inductive qualitative content analysis.

**Results:**

Our results showed a paradoxical situation for women with persistent health problems due to SPT. They struggled with their traumatised body, but healthcare professionals rejected their health problems as postpartum normalities. This paradox highlighted the women’s difficulties in accessing postpartum healthcare, rehabilitation, and sick leave, which left them with neglected healthcare needs, diminished emotional well-being, and loss of financial and social status. Our results indicated that these health problems did not diminish over time. Consequently, the women had to search relentlessly for a ‘key person’ in healthcare who acknowledged their persistent problems as legitimate to access needed care, rehabilitation, and sick leave, thus feeling empowered.

**Conclusions:**

Our study revealed that women with persistent SPT-related health problems experienced complex health challenges. Additionally, their needs for medical care, rehabilitation, and sick leave were largely neglected. Thus, the study highlights an inequitable provision of SPT-related healthcare services in Sweden, including regional disparities in access to care. Hence, the authors suggest that Swedish national guidelines for SPT-related care need to be developed and implemented, applying a woman-centered approach, to ensure equitable, effective, and accessible healthcare.

**Supplementary Information:**

The online version contains supplementary material available at 10.1186/s12913-024-11037-5.

## Background

Intrapartum and postpartum healthcare should ideally be high-quality, evidence-based, and a positive experience stemming from woman-centred care with a holistic approach based on human rights [1]. This approach acknowledges each woman’s articulated needs and expectations in her social, emotional, physical, spiritual, and cultural context [2]. Nevertheless, during the first year postpartum, about one in four Swedish women with severe perineal trauma (SPT) [3], i.e., a third- or fourth-degree perineal laceration involving the anal sphincter muscle and anorectal mucosa at vaginal childbirth [4], are dissatisfied with their care and one in three women report ongoing health problems related to their SPT. Women with SPT may suffer from various physiological and psychological consequences such as pain [5, 6]*,* incontinence [7], defecation problems [8], vaginal prolapse [5], sexual dysfunction [9] or depression and anxiety [10–12].

Reducing physical symptoms is essential to support emotional and social recovery after any perineal trauma [13, 14]. Women with SPT emphasise that professional, competent, and respectful attitudes from healthcare professionals (HCPs), including individual and adapted information, facilitate, and promote their postpartum recovery. Thus, the HCPs’ competence and knowledge of treatment options is a prerequisite for women to access needed care [15]. An additional problem in the Swedish context is the lack of national recommendations or guidelines, which enables each of the 21 regions to develop own regional and local guidelines. An audit of the existing regional and local guidelines for prevention and care of SPT shows an unexpected diversity or lack of evidence-based recommendations [16]. However, dissatisfaction with access to healthcare has been expressed by women with persistent, i.e., beyond one year postpartum, SPT-related health problems [6, 17]. Furthermore, women criticise inadequate or absent support [6, 18], poor information and education [6, 10, 18], and lack of follow-up care regarding SPT and its potential psychological and social consequences [6, 10]. Postpartum care focuses more on the baby than the mother’s well-being [18, 19]. Also, the available treatment options are perceived as limited and outdated by those with access to needed care [17, 18]. Moreover, women with SPT describe that some HCPs tend to normalise their SPT-related problems [10, 17–22], and women are met in unprofessional and disrespectful ways [17, 23], where HCPs are perceived as ignorant, nonchalant, and questioning women’s symptoms [10, 17]. Previous research [24] indicates an institutional objectification of women with SPT by Swedish healthcare providers hindering access to healthcare, sick leave, and occupational rehabilitation after SPT. In contrast, women also report being acknowledged and liberated when HCPs have a professional and empathic approach and provide continuity of care that enables access to care for persistent SPT-related health problems [17–19, 25]. Thus, several women who sustained an SPT during childbirth do not experience access to needed and necessary care, a fact that needs further exploration.

Globally, sexual and reproductive health and rights (SRHR) are crucial for individual health and gender equality [26]. Current issues within SRHR and midwifery are controlled by the institutional power in health institutions, i.e., medical power [27], connected to the still-existing economic and educational disadvantages of women globally, which are also feminist issues [26, 28]. As midwife stands for ‘with woman’ [28], gender or feminist approaches are used in advancing midwifery theory [27, 29] and various aspects of SRHR topics such as breastfeeding promotion [30], birth plans [31] and attitudes towards contraceptives [32]. In midwifery and feminist approaches, the biological material body and the socially constructed gendered body are viewed as intertwined [33]. Moreover, midwifery care is recommended to be woman-centred [1, 2], focusing on the individual woman’s needs and transferring control from the institution to the woman herself. However, despite the different organisations of sexual and reproductive healthcare between countries, international research shows similar results regarding women’s diverging experiences with postpartum SPT-related healthcare [6, 15, 17, 18].

In sum, there is growing evidence showing that many women with persisting health problems caused by SPT are often, but not always, met with mistrust and ignorance when seeking care for their problems. Even though there may be national, regional, or local protocols or guidelines for care after SPT, women with persistent SPT-related problems still raise their voices about the difficulty of getting access to competent quality care. This indicates a potential gender bias [34] and a need for gender theoretical perspectives in midwifery [28], as utilized in this study. Additionally, few studies explore the care-seeking experience among this group of women in a longer time perspective after childbirth when the SPT occurred.

## Methods

The aim of this study is to explore how women with self-reported persistent SPT-related health problems experience their contact with healthcare services 18 months to five years after childbirth when the SPT occurred.

### Study design and context

The present study is part of a larger research project investigating the long-term consequences of SPT on quality of life, working life, and healthcare contacts. This study had an inductive qualitative interview study design applying qualitative content analysis to analyse data [35–37]. This method searches for patterns, e.g., by identifying similarities and differences in the data. The researchers obtain an in-depth understanding of the studied phenomenon through abstraction and interpretation [36]; thus, an appropriate method to apply to capture women’s experiences of their healthcare encounters when seeking medical help and support. Throughout the research process, the recommendations for qualitative research according to ‘Consolidated criteria for reporting qualitative research’ (COREQ) were followed [38].

Sweden has 21 partly independent regions primarily responsible for providing healthcare services to the population. Healthcare services are tax-funded, and the regions have extensive autonomy to decide upon the healthcare services within each region based on the frameworks of the Health and Medical Service Act [39]. Additionally, within the Swedish social security system, 480 days of paid parental leave are allocated to each child in Sweden and can be utilised by their legal guardian(s) until the child is twelve years old. Of these 480 days, 60 days are specifically assigned to each parent, and the remaining days are split between parents as desired. The financial compensation is based on the parent’s income and is financed by taxes [40].

In Sweden, midwives are the primary care providers to women with normal pregnancies, births, and postpartum care. In case of complications to pregnancy and childbirth, midwives collaborate with other medical professionals, especially obstetricians. For example, midwives suture first- and second-degree perineal lacerations, while obstetricians are responsible for all SPT repairs [41]. Generally, in Sweden, women who sustain an SPT during childbirth are offered a check-up with the obstetrician responsible for the repair before discharge and should also have a follow-up with an obstetrician or sometimes a physiotherapist within the postnatal period. Thereafter, women with no mayor initial healing problems are advised to contact relevant healthcare services if any health issues related to the SPT should arise in the future. Women presenting with complicated healing are treated accordingly. Additionally, women with second- to fourth-degree perineal lacerations are assessed with questionnaires three times during the first year postpartum by the National Perineal Laceration Register. However, there are no recommendations in Sweden for prolonged check-ups for women with SPT after the postnatal period and no guidelines on organised check-ups for women with prolonged symptoms due to SPT exist [42, 43].

### Procedure

Women with persistent SPT-related health problems and characteristics were purposively recruited to achieve a heterogeneous sample reflecting multiple experiences. An overview of inclusion and exclusion criteria can be found in Table 1.

**Table 1:**
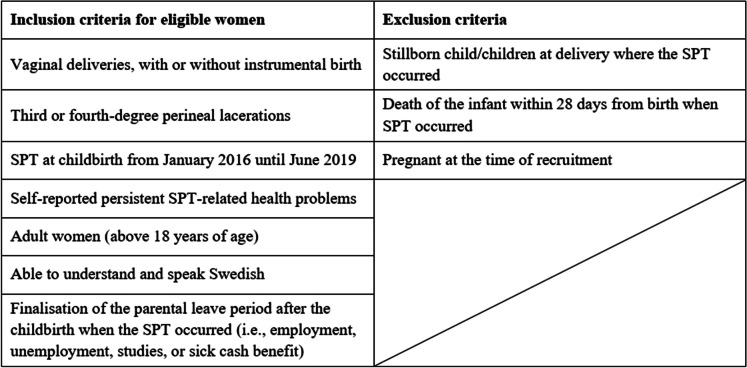
Overview of inclusion and exclusion criteria

The closed Swedish Facebook community ‘Förlossningsskadad? Du är inte ensam!’ [‘Injured at childbirth? You are not alone!’] functioned as a recruitment platform for a national sample of women reporting persistent SPT-related health problems. The Facebook community is secluded to women with SPT and started in 2014. During the data collection period (Nov 2020 – Feb 2022), the group had over 7,600 members; today, the community has grown to include over 9,500 members [44].

In late November 2020, the administrators of the Facebook community pinned a digital poster with study information and a link to the study homepage in the group feed. The study homepage contained written information on the research project and contact details for the research group if any women wanted additional information about the study. Interested potential participants contacted the research group via a contact form on the homepage, and the first author (KT) confirmed that the potential participants met the inclusion criteria via telephone. Thirteen participants from different parts of Sweden showed interest in participating and left their contact information. One woman never responded to our efforts to reach her. The remaining twelve women fulfilled the inclusion criteria and were invited to an interview. Before the interview, the women answered a digital survey on background data (such as demographic data, education, employment, sick leave, and childbirth history) distributed via REDCap^®^, a web-based application to create secure online questionnaires and research databases [45]. The interviews were finalised in February 2022.

### Data collection

We collected data via individual open-ended interviews [46], supported by a semi-structured interview guide [see Additional file 1]. The interview guide, developed by KT and MP with input from MW and IL, was based on literature reviews, our awareness of gender as a social construct [33], and the clinical pre-understanding within the research group. After a pilot interview conducted by KT (not included in the data), minor adjustments were made to the interview guide. The final interview guide covered the topics of everyday life experiences, work, and general functioning. However, despite the mentioned interview topics, the emergent study design and the ability to speak freely about what was perceived as important for their daily functioning, the contacts with healthcare services was brought up in vivid and extensive narratives by all participants as part of their descriptions of their challenges in everyday life and their ability to function at work. Hence, the experiences the women made of the healthcare services played an important role for the women in their daily management of SPT-related health problems.

As data collection occurred during the COVID-19 pandemic, all participants were interviewed digitally via Zoom^®^ [47, 48]. With the participant’s consent, the interviews were audio-recorded via Zoom^®^ and a separate digital recorder (as backup). Any Zoom video files automatically generated were deleted directly after the termination of the interview to protect participants’ identities. The first author interviewed all women; in two interviews, co-authors (IL or MW) also attended. The authors had no professional or personal affiliation with the enrolled participants*.* Detailed interviews ranging from 29 – 112 minutes (median: 61.5 minutes) gave extensive data. All interviews were performed in Swedish and transcribed verbatim. After that, the first author validated the transcripts for accuracy by reviewing the text while listening to the recordings.

### Authors’ pre-understanding and theoretical positionality

The research group comprises three midwives (KT, IL, MP) and one physiotherapist (MW). We all have extensive professional experiences from clinical practice in primary and in-patient care, where three authors (KT, IL, MP) have specific professional experiences of caring for women with SPT. Additionally, we are women, feminists, and mothers with various birth experiences. Further, the group holds expertise in gender studies and qualitative research within midwifery science, such as perineal trauma and medical sociology. Hence, we stem from a social constructivist research standpoint and utilise ourselves as co-constructors in the analysis process. As feminist researchers, we apply a gender theoretical lens to the data.

### Data analysis

The interviews were analysed using qualitative content analysis with an inductive and stepwise approach focusing on the manifest and latent content [35–37]. The interviews, transcripts, and analysis steps were performed in Swedish.

The analytical procedure started with reading the transcripts multiple times while highlighting text, meaning units, with content relevant to the aim of this study. Then, identified meaning units were condensed, focusing on preserving their core meaning and labelled with manifest codes [35–37]. Initially, KT coded one interview and triangulated those codes with the principal investigator (MP). KT then coded the rest of the interviews. In the next step, similar codes were clustered, forming subcategories based on the manifest content. Moving towards an interpretation of the content, categories were created by the abstraction of subcategories. This was done by KT and MP separately and then triangulated to identify significant concepts. Next, the preliminary categories and subcategories were triangulated with the whole research group until a consensus was obtained. To answer the question of ‘what?’ and ‘how?’ within the data, the latent content and thread of meaning were identified by clustering and abstracting the emerging findings to form subthemes and a theme [36, 37]. The emerging findings were also peer-reviewed and discussed at a research seminar. The finalisation of the analysis resulted in an overarching theme and four subthemes. The translation of categories, subthemes, theme and inserted citations from Swedish into English was performed as a last step. The translation and choice of words were discussed between authors (all knowledgeable in English) to reach a consensus and minimise translation bias.

During the coding process, the researchers used MAXQDA^®^ [49], a software for organising, transcribing, analysing, and visualising qualitative research data, and Microsoft Excel^®^ [50] as aids to organise the codes.

## Results

### Demographics of included participants

The background characteristics of the twelve participants in the final sample are presented in Table 2.

**Table 2:**
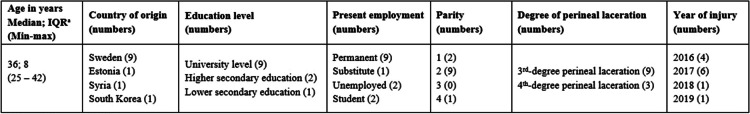
Demographic background data of the national sample of twelve women presented at group level

^a^Interquartile range (IQR)

The participants identified themselves as cis women, i.e., their gender identity matched their sex assigned at birth [51], and are thus referred to as ‘women’ in this paper. All women were in a partner relationship. The women reported a broad spectrum of physical and phycological health problems following the SPT at childbirth, e.g., urine or anal incontinence, pain in the lower abdomen, sexual dysfunction, and depression. Thirty per cent of the women had full-time employment, and the proportion of parental leave varied from 12% to 100% (three women had an ongoing parental leave with subsequent children at the interview). Further, 60% of the women had a sedentary occupation. Five women had been on sick leave after reconstructive surgery, and five reported sick leave for other reasons than their SPT.

The analysis resulted in one theme, ‘Overlooked by the obstetric gaze – living the paradox of a normalised but traumatised postpartum body’, with related subthemes ‘Questioning whether it’s all in my head’, ‘Fighting persistently for access and legitimacy in no (wo)man's-land’, ‘Facing multidimensional losses when no help in sight’, and ‘Depending on other’s advocacy to navigate an arbitrary system’. An overview of the findings is presented in Table 3. The findings are presented as an overarching theme and thereafter, the related subthemes and categories. Citations from the participants illustrate the findings. All women have been allocated pseudonyms in the result presentation.

**Table 3:**
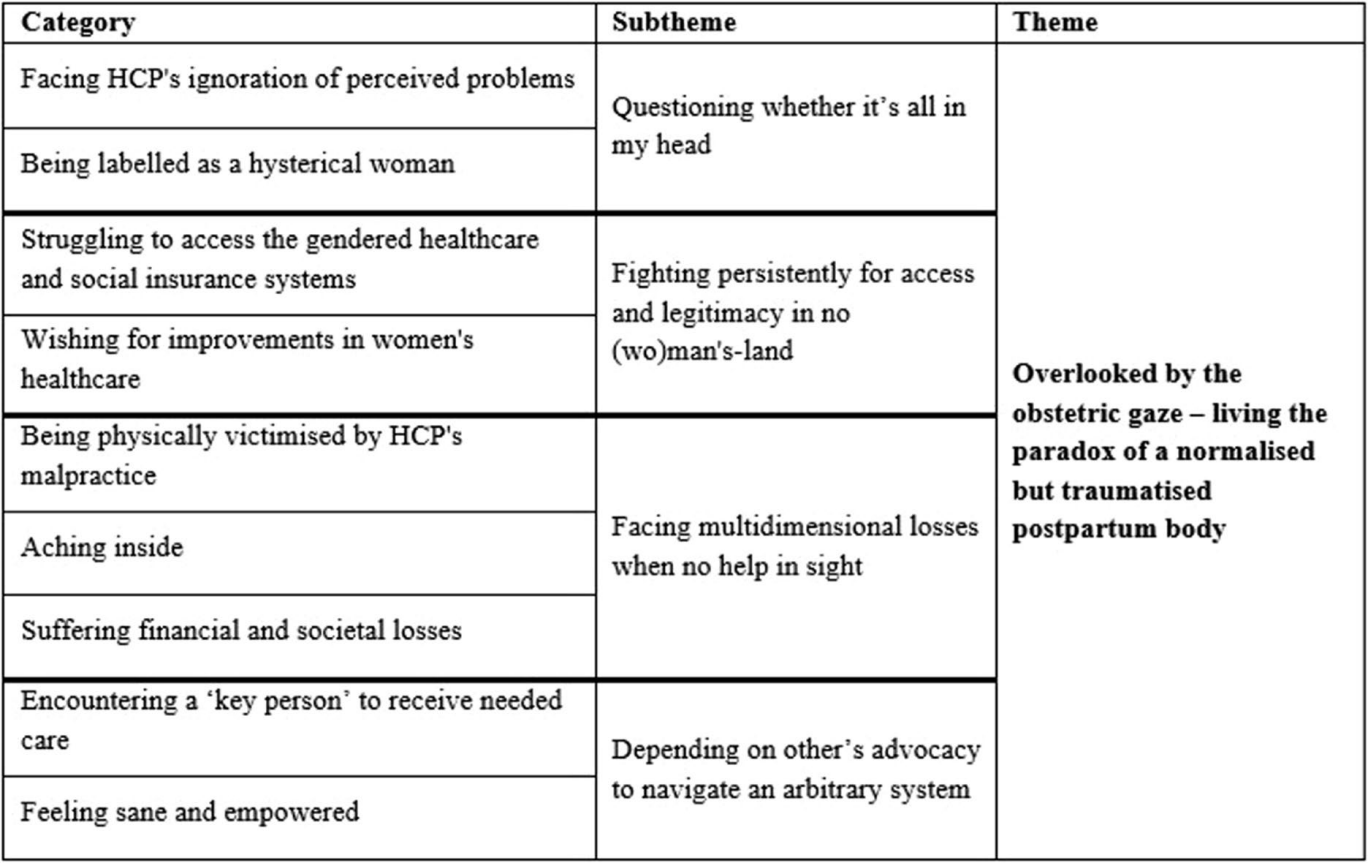
Overview of categories, subthemes, and theme

### Overlooked by the obstetric gaze – living the paradox of a normalised but traumatised postpartum body

The latent theme ‘Overlooked by the obstetric gaze – living the paradox of a normalised but traumatised postpartum body’ represented the women’s experiences of healthcare encounters covering HCPs’ diminishing attitudes towards women’s persistent SPT-related health problems and the women’s difficulties accessing healthcare and sick leave. We interpreted that the women were assessed by the HCPs’ ‘obstetric gaze’, i.e., a medical gaze in postpartum healthcare normalising their persistent health problems and judging the women’s lower abdomen as ‘fine’ by their looks. The obstetric gaze put the women in a paradoxical situation where HCPs normalised tangible symptoms to be a natural part of childbirth. With no medical legitimacy of the health problems, the women also felt labelled as ‘hysterical’ (exaggerating health problems) by the HCPs. As a result, on the one hand, they had to continue facing persistent and tangible health problems such as incontinence, pain or prolapses. On the other hand, no acknowledgement by HCPs of their health problems led them to question whether their problems were merely a product of their imagination and, thus, only existed ‘in their head’. The theme also comprised women’s struggle for legitimacy in a gendered healthcare system - a no-(wo)man's land. They experienced that healthcare services and social insurance systems were challenging to access and demanded a tenacious and extensive fight to obtain legitimacy for their health problems. Consequently, the women had to put up with neglected healthcare needs, negatively impacting their physical and emotional well-being, and financial and social status when no medical help or rehabilitation was available. However, some women had encountered an HCP who was empathic and understanding, hence not guided by the obstetric gaze. Such encounters legitimised persistent problems and were crucial for accessing needed care, sick leave, and rehabilitation.

#### Questioning whether it’s all in my head

The subtheme ‘Questioning whether it’s all in my head’ focused on the women’s experiences of facing ignoration and no confirmation of perceived health problems and thus being labelled as a hysterical woman. The related categories referred to a normalisation process that the women experienced in their encounters with HCPs, which made them question their bodily perceptions. Furthermore, the women felt accused of exaggerating symptoms because their persistent SPT-related health problems did not match HPCs’ views of acceptable postpartum symptoms. Thus, it could be understood that the women found themselves in a paradox of suffering from tangible physical consequences after SPT, which were normalised by HCPs and their ‘obstetric gaze’.

##### Facing HCP's ignoration of perceived problems

The women experienced the HCPs defining their persistent health problems after the SPT as ‘normal’. The HCPs assured the women that their problems would disappear with time or that transient motherhood-related aspects, such as breastfeeding or fragile vaginal mucosa, were the cause of the problem. One woman expressed:

“Then I felt, ‘It should not feel like this; this is something wrong’, and I sought medical attention and was seen by multiple physicians […] They thought my vaginal mucous membrane was not ready for intercourse. I was still breastfeeding, so they thought I should stop breastfeeding. Then maybe the mucous membrane would be restored, which was causing me the pain. I was not listened to at all. I was treated very poorly by one physician in particular, and despite second opinions and so on, nobody… nobody took me seriously.” (Linda)

Consequently, the women perceived that their concerns were ignored. They also learned that the HCPs saw their prolonged physical problems after SPT as an inevitable part of childbirth, which the women should accept. One woman resigned:

“But then [the physician] says something like this: ’Well, that's completely normal’, but I felt like, ‘Yes, but it doesn't feel normal'.” (Emma)

After the genital and pelvic floor examinations, the HCPs often guaranteed the women that ‘everything looked fine’, i.e., reinforcing the normality of the genital area. Although the women described to the HCPs that they struggled with SPT-related problems, their concerns were met with a comment on the physical appearance rather than a comprehensive examination of the pelvic floor's functionality.

One woman responded:

“They think ‘everything looks fine’ and ‘everything looks good and repaired’. I still have problems. I was also referred to a surgeon, who did a rectoscopy, and ‘it looked so nice’. Then, I was referred to a urotherapist to learn how to pinch my muscles because ‘everything would be so good’. She helped me get a second opinion in XX [town], where they discovered that there was still damage." (Jin)

Another woman expressed:

”I couldn’t care less what it [genital area] looks like. Nobody will be down there watching. I only need it [genital area] to function as intended.” (Anna)

Consequently, the women felt ignored and unheard in their contact with healthcare services. They perceived that HCPs did not listen to them, leaving them feeling invisible, sometimes even having severe health problems.

“I was hospitalised with sepsis before someone listened to me.” (Josefin)

##### Being labelled as a hysterical woman

The women also experienced being labelled as the ‘hysterical woman’ who exaggerated their persistent symptoms and had mental health problems. The women described how the HCPs accused them of imagining their SPT-related health problems. One woman indignantly revealed that the HCP she encountered said, 'These problems only exist in your head’ (Joanna), i.e., suggesting that the perceived symptoms did not exist and rejecting the health concerns. Hence, this attitude made some women believe their problems were a product of their imagination and sometimes made them even question their sanity.

Moreover, the HCPs’ condescending attitudes towards the women made them feel dismissed and devalued. For example, the women shared that HCPs laughed at them or were rough or cold during the examination. Moreover, HCPs expressed that they had ‘seen worse’ (Amanda). Some women also conveyed that they were advised ‘to drink some wine to feel better’ (Elin) when discussing painful intercourses due to their SPT-related health problems.

“You are constantly dismissed, ‘No, but everything looks fine, you have no problems’. Then you start to think you’re imagining things. And then you may not dare to talk about the injuries.” (Jin)

#### Fighting persistently for access and legitimacy in no (wo)man's-land

The subtheme ‘Fighting persistently for access and legitimacy in no (wo)man's-land’ referred to the women’s experience of gender constructs related to inaccessible healthcare services and their often year-long struggles to access this gendered healthcare and linked social insurance systems. The difficulties in accessing care created negative attitudes towards the healthcare services, making the women wish for general improvements in women’s healthcare.

##### Struggling to access the gendered healthcare and social insurance systems

The women pointed out that after giving birth, they needed more extensive information on their injury, precautions, available help (follow-up care or re-operation), and sick leave. To overcome the lack of required information, they had to request or actively search for it on their own, which also led to uncertainty about where and when to seek further help if needed.

“I was sent home with a brochure and a pat on the shoulder.” (Amanda)

The women also experienced a lack of adequate healthcare services targeted at their SPT-related health problems. For example, many women did not have access to a pelvic floor clinic or had to travel long distances to see specialists. Hence, their place of residence decided the quality of care the women received. Moreover, some women problematised the organisation of postpartum care as they missed out on follow-up care and even, in some cases, were denied follow-up care or referrals to specialised care were lost. As a result, some women had no opportunity to talk to the operating physician or experienced no follow-up care, although they requested it.

“They said it can take up to a year to get better. So, when that year had passed, and before starting to work again, I called different places in the hospital and asked: What should I do now? […] It took several months before I got an appointment with the surgeon for an assessment. And then I had to get a second opinion. So, it took like seven months before I got an appointment at [a specialist clinic].” (Hawa)

For the women, access to healthcare services, sick leave certificates, and HCPs’ dismissive attitudes were perceived as gender-related, i.e., difficulties in obtaining help from women’s healthcare services would not exist if the services were more women-oriented. One woman illustrated this by expressing: ‘If men gave birth to babies, the situation would not be like this’ (Joanna). Moreover, they perceived that women’s healthcare services were not prioritised. They explicitly stated that the absence of sick leave certificates and benefits was related to their gender. The women were expected to cope without sick leave benefits because vaginal and perineal lacerations of any scope were viewed as a natural part of childbirth, a normal process of a woman’s body. Thus, sequelae thereof did not exist or were taboo in society.

“Everything that happens during and after childbirth and related injuries has been a taboo discussion topic, so it has been completely ‘normal’ to suffer from persistent pain.” (Anna)

Another woman expressed:

“I have applied for compensation from the national patient insurance. I got rejection after rejection; nothing has gone wrong. I was told: 'You simply must expect these things in childbirth. And a caesarean section is not less risky'.” (Hawa)

Thus, the women argued that society and the government did not invest needed resources in women’s healthcare. In addition, those few women receiving a short period of sick cash benefits had it immediately after giving birth or after re-operation, but not for prolonged problems. Further, the women noted that they were not offered sick leave certificates due to persistent physical SPT-related health problems but instead due to mental issues, such as depression or anxiety.

“I've heard about women who have been mentally unwell and have hurt their children. So maybe physicians get cautious and put women on sick leave if they say, ‘I'm not feeling mentally well’. Then they act quickly because they think it's so important. But they don't think about the physical injuries because that's part of [childbirth].” (Jenny)

However, the women shared how they fought long and hard for acknowledgement and care and made demands; for many, this process had covered years. They had to repeatedly insist that something was wrong and felt pressure to prove their health problems to the HCPs. In some women, this led to their persistent problems being diagnosed and acknowledged after several years of delay. The struggle for care involved countless visits and referrals to different HCPs, demanding much strength and persistence, which exhausted them. Sometimes, the sequelae had to develop into an acute health situation, or some women decided to pay for private care to access the proper treatment and rehabilitation. Further, with time, they also became explicit about their demands for sick leave certificates and benefits.

“Well, it [short sick leave period because of birth traumas] just feels like scorn. To me, it is not a sufficient length of sick leave.” (Elin)

##### Wishing for improvement in women's healthcare

The perceived lack of adequate care and rehabilitation, access to sick leave benefits, and HCPs’ attitude negatively influenced the women’s opinions on healthcare services, especially postpartum healthcare. In addition, the women perceived many HCPs as unprofessional, indifferent, and unstructured. As a result, the women mistrusted the HCPs and lost hope in healthcare services. Thus, they were reluctant to seek further care and were anxious about receiving proper treatment or that HCPs would miss important things.

“I am not being listened to in women's healthcare. This is partly why I feel so disappointed.” (Linda)

“You just don't trust the healthcare system. […] Some people have been struggling with their injuries for like 18 years. But the [specialist clinic] – I finally received fantastic treatment, and what if it could be available everywhere [in Sweden]?” (Hawa)

Moreover, the women described a struggle for their rights when deciding whether to report the HCPs to the authorities and pointed out the need to improve women’s healthcare. Reporting HCPs was perceived as complicated as the women did not want to blame specific individuals. The women saw that the major problem lay within the healthcare system and with individual HCPs.

“In the end, I met a fantastic person [healthcare professional]. She wanted me to report the mistreatment when I eventually had the strength. Because no one listened when I said I was ill. So, she has offered to help me if I want to, but I don't know if I have the strength to file a complaint.” (Josefin)

A wish to improve women’s healthcare services was articulated, especially regarding personal follow-up care beyond one year postpartum and the possibility of full-time or part-time sick leave certificates and benefits for persistent problems on equal terms. This wish also strengthened their decision not to give up searching for help and to raise their voices to help themselves and other women.

“I received physiotherapy and the follow-up surveys [the Perineal Laceration Register] during the first year, but thereafter I would have liked to have an annual follow-up for the next years to ensure the status and potential re-operations. […] I can google, but I want to have that information in dialog with a living person, but you do not get that.” (Jenny)

Partaking in developing educational material for HCPs or starting a career within women’s healthcare were some women’s ways to contribute and increase competency in persistent SPT-related health problems.

“One of my strategies since I got the injury is also to try to influence. Being able to be involved and influence what postpartum care should consist of.” (Jin)

#### Facing multidimensional losses when no help in sight

The subtheme ‘Facing multidimensional losses when no help in sight’ covered physical and mental health consequences and the financial and social losses the participating women faced when no support or access to needed care and rehabilitation was provided.

##### Being physically victimised by HCP's malpractice

The women’s experiences covered either being misdiagnosed during the suturing after birth or in the following years when seeking help for persistent SPT-related health problems. Further, they shared how physicians had incorrectly sutured vaginal and perineal muscles after childbirth, leading the women to live with incontinence, pain, prolapses, or sexual dysfunction if their vaginas were sutured too tight. They also described how they endured infections, wound ruptures, sepsis, necrosis, and re-operations. Additionally, the women perceived a general lack of competency regarding communication and persistent SPT-related health problems, including problems related to sex life and sexual functioning, besides a more specific lack regarding suturing techniques and ultrasound examinations.

“I was referred to a specialist clinic. And they found out that all the muscles were separated, the internal and external sphincters were torn, and my pinching ability was kind of weak. So, it was quite the opposite, really, quite the opposite. None of what the other physician had said was true [laughs]. Absolutely incredible. And she is supposed to be a specialist.” (Hawa)

##### Aching inside

Living with troubled postpartum bodies and the absence of HCPs’ legitimation of the women´s problems made them struggle mentally, feeling speechless and silenced. This neglect reinforced irritation, anger, distress, bitterness, and disappointment towards the HCPs and the healthcare services. One woman illustrated the emotional struggle in this way:

“It's just that the health services don’t believe you, which makes you feel terrible. It's a big deal that no one listens.” (Josefin)

Moreover, the women felt uncertain about their health status due to a default medical diagnosis with concerns for their future and which staff to trust. Consequently, some had to bite the bullet, put up with their situation, and try to think positively. Other women were denying or diminishing their SPT-related health problems, accepting that their symptoms would improve, even disappear or that their condition was ‘normal’ as they had been told. Further, the women described despair because their neglected health problems caused by their SPT made them feel exposed, unsure, and hopeless. In some, this desperation resulted in a mental breakdown, a fear of losing custody of their child due to mental illness or suicidal thoughts.

“Something broke inside of me that day. I felt entirely omitted; I was close to leaving my son and committing suicide. Nobody understood how bad everything was.” (Elin)

Additionally, the women suffered emotionally when motherhood was crushed. Their partner had to take the primary responsibility for the family, and the children had to come in second place as the mothers suffered from various physical and mental health problems. As a result, the women felt they missed their children’s development and could not use their parental social security benefits as desired.

“I feel devasted because people tell me, ‘You are on maternity leave’. I’m not on maternity leave; I’m sick. I should be on sick leave.” (Jaanika)

##### Suffering financial and societal losses

Moreover, the women suffered financially and societally due to persistent health problems. Some women were denied financial compensation from Patient Insurance (a national insurance system where patients can seek compensation for care injuries). The Social Security Agency and the HCPs were perceived as obstacles to receiving sick cash benefits. They noted that ‘extensive’ health problems were required to receive sick cash benefits and that their health problems paradoxically were not seen as extensive or even a problem per se by the HCPs; hence, no sick leave certificates were issued.

“He [the physician] tried to argue and clarify my pain situation in the sick leave certificate to meet the requirements for a sick leave benefit at the Social Security Agency. I was in so much pain and had to lie down to breastfeed. But, no, ‘If you can manage to hold the baby when breastfeeding, then you are on maternity leave, not sick leave benefit’ [mimicking the official at the Social Security Agency who rejected the certificate and consequently also the sick cash benefit]”. (Jaanika)

Furthermore, the women were set back financially and societally because they could not work full-time due to their persistent health problems. Therefore, some women chose to compensate for their work absence with part-time parental benefits to diminish their working hours and cover their inability to work due to persistent SPT-related health problems. Without a sick leave certificate, i.e., the physicians or the officials at the Social Security Agency’s acknowledgement of a ‘true’ health problem, partners or other relatives were obliged to adjust their work schedules to support or unburden the woman’s suffering and inability to work full-time. This reduction in working hours for the SPT-affected women and, in some cases, their partners was expressed to potentially negatively affect their upcoming careers and pensions. As a result, the women experienced being caught between stools in the social insurance systems:

“[…] You end up in a position where you are neither on sick leave nor unemployment benefits and at the same time cannot perform any offered work [due to persistent problems]. But multiple societal bodies demand and expect you to be a part of the working force, and nobody really listens.” (Elin)

#### Depending on other’s advocacy to navigate an arbitrary system

The last subtheme, ‘Depending on other’s advocacy to navigate an arbitrary system’, highlights the women’s experiences of, often by chance, finding a single devoted professional, i.e., a ‘key person’, to access needed care and rehabilitation. Such a ‘key person’ was vital to recognising persistent problems, legitimating symptoms, and enabling access to needed care, sick leave, and rehabilitation. The women who finally had legitimation for their health problems described that the medical diagnosis also came with a feeling of sanity and empowerment, relieving them of their paradoxical situation.

##### Encountering a ‘key person’ to receive needed care

A support system was a prerequisite for enduring their health problems and finding the strength to fight for access to care. This system could be a partner, other family members, or friends who gave the women power and courage, but most importantly – encountering a professional who saw their problems and provided referrals or other options to obtain the needed help and support. In most cases, women would search for years for competent HCPs, such as midwives, physicians, or physiotherapists, who would listen and acknowledge persistent problems. This ‘key person’ showed empathy and trustworthiness, creating relief and security. Further, the ‘key person’ was portrayed as competent, attentive, professional, and respectful. The ‘key persons’ also shared women’s outrage at the mistreatment and default healthcare they endured. Additionally, these ‘key persons’ were surprised that the women were not on sick cash benefits due to their symptoms and that they had to compensate for their financial situation with parental benefits or reduced working hours and lower salaries. Consequently, finding this ‘key person’, often by chance or word of mouth, was crucial for accessing care and marked a significant turning point in the women’s recovery.

“I sought help from another midwife, as I felt something was wrong. This midwife referred me to the physiotherapist, who referred me to a specialist, who then referred me to surgery and rehabilitation.” (Malin)

Some women received follow-up care for their persistent SPT-related health problems during the first year postpartum. If persistent problems occurred and were acknowledged, the women were offered different surgical approaches with various outcomes, consultations by colon specialists, physiotherapy, and psychiatric care. They were grateful for the help they received but felt more comprehensive care was needed.

##### Feeling sane and empowered

Confirmation of persistent SPT-related health problems was expressed as liberating, strengthening and, as one woman put it, a ‘win’ (Elin). Receiving a medical diagnosis and appurtenant treatment was relieving because the medical confirmation of the symptoms released a considerable burden. These women described being acknowledged, and the diagnosis proved that health problems existed, and the struggles were not in vain. Furthermore, it explicitly stated to everyone, including themselves, that they were not ‘crazy’, ‘imagining things’ or ‘hysterical’.

“So, my laceration has been classified as an injury caused by the healthcare services. This was somehow a confirmation. It's not just that it's in my head, but it has been established that it is a medical injury, and it could have been avoided.” (Jin)

Alongside feelings of sanity and being legitimised, the women experienced empowerment. The women felt supported and confident. Thus, finding an agency to address the taboo of their SPT by talking openly about it and helping others in the same situation was also seen as therapeutic. Further, the legitimation of the sequelae and access to appropriate care gave them time to heal and process their trauma. Receiving sick leave certificates and benefits was seen as a part of the empowerment and legitimacy of their persistent SPT-related health problems, reducing stress, and easing the financial burden. Furthermore, access to occupational rehabilitation and understanding at work became available. Thus, the women who had received the help they needed after a struggle to obtain it were hopeful about the future and possible recovery.

“I have regained my authority to speak up. It [SPT-related health problems] should be out in the open, not withheld.” (Jaanika)

## Discussion

Our main finding was that women with persistent health problems due to SPT at childbirth were caught in a paradox of living in a normalised but traumatised body, and their health problems were rejected as postpartum normalities. Furthermore, our results elucidated the difficulties in accessing postpartum healthcare, rehabilitation, and sick leave benefits. Therefore, the women struggled with neglected healthcare needs, diminished emotional well-being, and loss of financial and social status. Our study highlighted experiences up to 5 years after sustaining SPT, which showed that some women’s SPT-related health problems do not diminish with time. They faced challenges functioning in daily life, at work, and in society. In contrast, finding a ‘key person’, i.e., a professional who acknowledged the women’s persistent problems as legitimate, was a prerequisite for accessing all the needed care and sick leave and enhancing empowerment for the women. Thus, this ‘key person’ was not blinded by the obstetric gaze and instead used their agency and advocacy as support.

In the following, we will discuss our findings related to other empirical studies and problematise them with theoretical reflections.

### The paradox of normalising the postpartum body

In our findings, the paradox arose when the HCPs dismissed physical health problems after SPT despite women’s perceived symptoms. Central in this context was a normalisation process where health problems were regarded as ‘normal’ by HCPs, a phenomenon also found in prior research on SPT [17–22]. The HCPs’ normalisation of women’s health problems can also be found regarding other medical conditions affecting women, such as pelvic organ prolapse [52], menstrual pain [53], endometriosis [54] or nausea and vomiting during pregnancy [55]. In light of the medicalisation of women’s healthcare, where the medical field has sought to pathologise natural bodily processes such as pregnancy and childbirth [33], actual medical conditions such as persistent SPT-related health problems are paradoxically normalised. Our findings, therefore, highlight the need to challenge HCPs’ views of what constitutes a ‘normal postpartum body’ or ‘normal postpartum symptoms’ after sustaining SPT.

### The key to healthcare

In the context of denied legitimacy of health problems and neglected needs, it appeared that the women became dependent on the goodwill of a ‘key person’, personified as the respectful, competent, and empathetic HCP. Prior research on SPT has also found women struggling with accessing healthcare [6, 17] and specific HCPs as enablers of care [12]. The dependency on a ‘key person’ to access adequate care might highlight a structural problem within the provision of postpartum SPT-related healthcare. Globally, there are a few national guidelines on SPT management and prevention [56]. Additionally, no national guidelines regarding postpartum care of SPT exist in Sweden, and pelvic floor teams are only available in some Swedish regions [16]. In our study, the women lacked information, and competent HCPs were hard to find or located far away. Other studies have shown poor patient information and education as a postpartum problem [6, 10, 18], indicating a need to develop targeted oral and written information on wound healing and recovery. Further, women in Australia describe similar challenges to accessing SPT-related healthcare when having persistent SPT-related health problems [18]. The absence of national Australian guidelines may have led to inconsistent care, failing to meet women’s healthcare needs. Further, women from rural areas have had additional difficulties accessing needed care. In 2021, a clinical standard for SPT was implemented in Australia, comprising care standards for follow-up [57]. Thus, to improve the national situation in Sweden, more research and resources must be allocated to develop evidence-based recommendations, preferably internationally accepted guidelines [56]. Moreover, the accessibility of SPT-related healthcare, such as pelvic floor clinics, needs to be expanded so that women can easily meet their ‘key person’ if required.

### Woman-(de)centred care?

We found that HCPs were obstructed by their obstetric gaze when assessing women with persistent SPT-related health problems. Obstetric gaze derives from the medical gaze notions [58], suggesting a gaze that splits the individual from the body, constructing the care-seeker as a medical object or condition instead of an individual with a social context. This gaze blinded HCPs who normalised obvious health problems. Recent advances in women’s healthcare in industrial countries and midwifery research show development towards continuity of care models with a woman-centred approach in different caseload-midwifery projects and informed choice regarding place of childbirth [28, 59–61]. Wom**e**n-centred care [2] is a widespread care philosophy within midwifery that advocates for providing individualised care to women. Further, wom**a**n-centred care emphasises the individual woman’s healthcare needs and situation, incorporating the concepts of choice, control, continuity of caregiver, and self-determination. It can be argued that the obstetric gaze obstructed HCPs in providing wom**a**n-centred care because they did not acknowledge the women’s healthcare needs. Consequently, the women did not have control over their health situation. Making women feel empowered [2, 62] is crucial in woman-centred care. Hence, the ‘key persons’ in our study managed to provide wom**a**n-centred care where acknowledgement of problems as real medical problems and access to care made the women experience empowerment. Therefore, we argue that guidelines regarding follow-up care after SPT should ideally be developed with wom**a**n-centred care as its core.

### Everything looks fine

The biomedical model has traditionally focused on normality and abnormality rather than health [63]. Theoretically, the ‘obstetric gaze’ is closely tied to the ‘medical gaze’ and the ‘male gaze’, referring to the biomedical paradigm and its power [27, 58]. In our study, the obstetric gaze judged the women’s persistent health problems due to SPT as ‘normal’ and the appearance of their genital area as ‘fine’, which created a paradoxical situation regarding the legitimacy of their ongoing health problems after SPT. Generally, the healthcare sector is critiqued for reducing the body to only incorporating organs and tissue, i.e., focusing on physical symptoms [27].

The women in our study, of which most showed more than one significant symptom after SPT, noted that HCPs would comment on the physical appearance of the perineal area rather than its functionality by telling them that ‘everything looked fine’. The focus on looks rather than functionality regarding SPT-related health problems aligns with the findings presented by others [17]. Having women describe how their persistent physical pelvic floor problems after SPT during childbirth are trivialised, normalised, questioned, and labelled as mental health issues is of utmost concern. This implies the need for rapid improvements in HCPs’ knowledge and organisation of care but also raises the question of what is considered a normal status and recovery after any perineal laceration in the short- and long-term perspective. A similar discursive focus on women’s appearance instead of their health problems has also been found among HCPs when women seek care for chronic pain [64]. The sentence ‘Everything looks fine’ can be interpreted as an objectifying, gendered discourse in an obstetric context. This discourse may reinforce the obstetric gaze and, in the broader sense, the medical gaze [58]. The Swedish Health and Medical Care Act [39] advocates for the respectful treatment of patients. Hence, it is noteworthy that the women experienced being judged by the looks of their genital area in their medical encounters rather than HCPs addressing the functionality. Such treatment does not align with the legislation and calls for a discourse analysis of the attitudes of HCPs towards women with persistent SPT-related health problems and their experiences of providing care for affected women.

### Being subjected to obstetric gaslighting

In light of the women’s perception of their dismissal as dramatic, illegitimate, and irrational patients, we argue that they faced so-called ‘gaslighting’ in an obstetric context [65, 66]. Thus, the women experienced being offered sick leave for mental problems instead of their perceived physical health problems, depicting them as hysterical women who exaggerated their condition. Gaslighting is a concept used in medicine in general [66] and in obstetrics regarding traumatic childbirth experiences [65]. The concept of hysteria, i.e., a prior medical diagnosis and historical concept theoretically linked to femininity [67, 68] and ‘obstetric gaslighting’ [65], has also been found in research on women’s chronic pain [64] and endometriosis [69]. Men with chronic pain are perceived as brave, and women in pain are hysterical, emotional, whining, malingering, or imagining pain [64]. Further, women with endometriosis are viewed as ‘reproductive bodies’ with a proneness for hysteria [69]. Obstetric gaslighting, enforced by the normalisation of SPT-related health problems and the gendered stereotype of women as hysterical patients, puts women with SPT in an inferior position towards HCPs and can, therefore, be interpreted as a demonstration of institutional power [65]. Hence, being overlooked by the obstetric gaze might constitute a form of obstetric gaslighting, a concept that has not been applied to SPT before.

### Implications and significance

Our study indicated that women continue to have problems accessing healthcare for persistent SPT-related health problems several years postpartum. Additionally, women with persistent SPT-related health problems often depended on a ‘key person’ with the competence to open the doors to comprehensive care, as shown in our findings. The Swedish Government launched a multi-million project from 2015 to 2022 to improve and promote women’s health [70]. Despite this investment, the depicted experiences of the included women reflect upon remaining structural and clinical problems within Swedish healthcare, which need further attention, investigation, and actions. Additionally, there are considerable differences in reported satisfaction and prevalence of complications at the one-year follow-up between the regions [3], indicating that there are suboptimal healthcare services. With a significant variation in satisfaction and recovery at one year, there are reasons to believe that women with prolonged problems may experience problems getting access to needed care.

Our study also showed that SPT-related healthcare services are not available on equal terms to women with persistent SPT-related health problems. In general, many women within this group had problems accessing care and sick leave for years. However, depending on where the women reside, not all women have access to specialised care. This inequity may be explained by Sweden having 21 self-governing health regions, and in the absence of national guidelines regarding SPT care and follow-up, the healthcare provision for affected women varies. To secure access to postpartum care for women with SPT in general and those with different prerequisites within this group, implementation studies are needed to develop and evaluate the effect of national guidelines for follow-up care regarding SPT.

### Strengths and limitations

This study has strengths and limitations that need to be addressed. A significant strength, enhancing credibility and transferability, was providing a clear context and thick descriptions of our results, where we thoroughly portrayed the women’s voices using quotations [35]. Further, our detailed account of the study context, data collection, and data analysis process facilitated the transferability of our study. Including three women born outside of Sweden added to the variety of the sample and thus improved credibility because qualitative research often overlooks immigrants' experiences. However, the migrant women spoke Swedish well enough to participate in an interview, indicating that they have been living in Sweden for some time and might be familiar with the healthcare system. Finally, the credibility and dependability of this study were also strengthened by the frequent use of interdisciplinary triangulation between the authors throughout data analysis and the writing process, as well as peer review at a research seminar.

A potential limitation was that this study may not have fully explored the situation of women with fourth-degree lacerations or those with lower education, as most participants had third-degree perineal lacerations and higher education. Further, we could not include non-binary persons and same-sex or single parents, which may be a weakness; consequently, future studies should focus on the under-represented participant groups and migrant women needing an interpreter. Additionally, all women responded voluntarily to the study invitation. Thus, our participants might be particularly outspoken about their problems or interested in raising their voices or experiences. However, they represented a variety of persistent SPT-related health problems of various severity, and some had been able to get access to medical help, whereas others had not. Additionally, our findings cohered to similar studies [12, 17] covering shorter periods after the SPT, which may indicate that the experiences of the challenging search for needed help remain over time. Therefore, our findings may reflect other women’s experiences seeking care for SPT-related health problems and may be transferable to other women’s experiences with persistent health problems of a rare condition.

The data for this study was comprehensive and rich. Information power in qualitative research is an ongoing discussion, and the number of participants and their representativity can be seen as a limitation of credibility and transferability [71, 72]. Graneheim, Lindgren and Lundman [36] argue that sample size should be determined by the study’s aim and the data’s quality so that variations in experiences can be captured. They do, therefore, not recommend a specific number of participants, but others do [71]. With this in mind, the authors believe that the women’s detailed descriptions of the included concepts and the extensive length of the conducted interviews enabled us to achieve sufficient information power based on the richness of the data [72].

## Conclusions

By qualitatively exploring how women with persistent SPT-related health problems experienced their healthcare encounters, we interpreted that they faced a paradox of being reassured of normality by HCPs despite reporting sequelae symptoms. Thus, women’s needs for medical care, rehabilitation, and sick leave were largely neglected. Further, our study might indicate a structural problem within women’s postpartum healthcare, indicating that access to care depended on encountering a ‘key person’, a professional who acknowledged persistent problems as real symptoms. Access to quality care provided with a professional attitude was essential for the future well-being of women with persistent SPT-related health problems. Thus, it should not depend on meeting a single ‘key person’. Therefore, national guidelines for long-term postpartum care of persistent SPT-related health problems must be developed in Sweden. Additionally, to ensure that healthcare services meet the individual needs of women with persistent SPT-related health problems, it is crucial to consider arranging the organisation and availability of quality care for these women from a woman-centred perspective.

### Supplementary Information

Additional file 1. Semi-structured interview guide for individual interviews; contains interview questions aimed at highlighting the experience of everyday life and working life after suffering 3^rd^ or 4^th^ degree perineal laceration at childbirth (i.e., severe perineal trauma [SPT]).

Additional file 2. Consolidated criteria for reporting qualitative studies (COREQ): 32-item checklist.

## Data Availability

The original recordings and transcripts from the current study are not publicly available due to securing the individual privacy and confidentiality of the participants. Data are available from the corresponding author upon reasonable request.

## Abbreviations

HCPs: Healthcare professionals
IQR: Interquartile range
SFO-V: Strategic Research Area Health Care Science
SPT: Severe perineal trauma
SRHR: Sexual and reproductive health and rights
WHO: World Health Organisation

## References

[1] World Health Organization (WHO). WHO recommendations: Intrapartum care for a positive childbirth experience. Geneva: WHO; 2018. Cited 2024 Feb 14. Available from: https://www.who.int/publications/i/item/9789241550215. 30070803

[2] Leap N (2009). Woman-centred or women-centred care: does it matter?. Br J Midwifery.

[3] Uustal E. Bristning vid förlossning grad 3 - 4. Årsrapport från GynOp-registret avseende operationer utförda år 2022. [Laceration at childbirth, grade 3 - 4, Annual Report of surgeries 2022]. Umeå: The Swedish National Quality Register of Gynecological Surgery; 2023. Cited 2024 Maj 8. Available from: https://www.gynop.se/rapporter/arsrapporter/.

[4] World Health Organization (WHO). International statistical classification of diseases and related health problems (ICD) 10th revision. Geneva: WHO; 2019. Cited 2024 Maj 8. Available from: https://icd.who.int/browse10/2019/en.

[5] d’Almeida I (2020). Women’s experiences following obstetric anal sphincter injury. J Pelvic Obstet Gynaecol Physiother.

[6] Darmody E, Bradshaw C, Atkinson S (2020). Women's experience of obstetric anal sphincter injury following childbirth: An integrated review. Midwifery..

[7] LaCross A, Groff M, Smaldone A (2015). Obstetric anal sphincter injury and anal incontinence following vaginal birth: a systematic review and meta-analysis. J Midwifery Women's Health.

[8] Samarasekera DN, Bekhit MT, Wright Y, Lowndes RH, Stanley KP, Preston JP (2008). Long-term anal continence and quality of life following postpartum anal sphincter injury. Colorectal Dis.

[9] Andreucci CB, Bussadori JC, Pacagnella RC, Chou D, Filippi V, Say L (2015). Sexual life and dysfunction after maternal morbidity: a systematic review. BMC Pregnancy Childbirth.

[10] Beck CT (2021). Effects of fourth-degree perineal lacerations on women's physical and mental health. J Obstet Gynecol Neonatal Nurs.

[11] Keighley MR, Perston Y, Bradshaw E, Hayes J, Keighley DM, Webb S (2016). The social, psychological, emotional morbidity and adjustment techniques for women with anal incontinence following Obstetric Anal Sphincter Injury: use of a word picture to identify a hidden syndrome. BMC Pregnancy Childbirth.

[12] Lindqvist M, Lindberg I, Nilsson M, Uustal E, Persson M (2019). "Struggling to settle with a damaged body" - A Swedish qualitative study of women's experiences one year after obstetric anal sphincter muscle injury (OASIS) at childbirth. Sex Reprod Healthc.

[13] Shoorab NJ, Taghipour A, Mirteimouri M, Roudsari RL (2020). Social recovery: a neglected dimension of caring for women with perineal trauma in Iran. Iran J Nurs Midwifery Res.

[14] Jahani Shoorab N, Mirteimouri M, Taghipour A, Latifnejad Roudsari R (2019). Women's experiences of emotional recovery from childbirth-related perineal trauma: a qualitative content analysis. Int J Community Based Nurs Midwifery.

[15] Swedish Agency for Health Technology Assessment and Assessment of Social Services. Practices to improve detection of perineal tears and women’s views and experiences of healthcare providers following sustained perineal tear. A systematic review. 2021. Report No.: 323. 36170472

[16] Persson M, Lindberg I, Ohman A (2023). Regional and clinical guidelines for prevention and care of obstetric anal sphincter injuries - A critical frame analysis. Midwifery.

[17] Huber M, Tunon K, Lindqvist M (2022). "From hell to healed" - A qualitative study on women's experience of recovery, relationships and sexuality after severe obstetric perineal injury. Sex Reprod Healthc.

[18] Priddis HS, Schmied V, Kettle C, Sneddon A, Dahlen HG (2014). "A patchwork of services"–caring for women who sustain severe perineal trauma in New South Wales–from the perspective of women and midwives. BMC Pregnancy Childbirth.

[19] Priddis H, Schmied V, Dahlen H (2014). Women's experiences following severe perineal trauma: a qualitative study. BMC Womens Health.

[20] Williams A, Lavender T, Richmond DH, Tincello DG (2005). Women's experiences after a third-degree obstetric anal sphincter tear: a qualitative study. Birth.

[21] Herron-Marx S, Williams A, Hicks C (2007). A Q methodology study of women's experience of enduring postnatal perineal and pelvic floor morbidity. Midwifery.

[22] Crookall R, Fowler G, Wood C, Slade P. A systematic mixed studies review of women’s experiences of perineal trauma sustained during childbirth. J Adv Nurs. 2018;74(9):2038–52. 10.1111/jan.13724 29791012

[23] Priddis H, Dahlen H, Schmied V (2013). Women's experiences following severe perineal trauma: a meta-ethnographic synthesis. J Adv Nurs.

[24] Tjernström K, Lindberg I, Wiklund M, Persson M (2023). Negotiating the ambiguity of an (in)authentic working life: a grounded theory study into severe perineal trauma. BMC Women's Health.

[25] Lindqvist M, Persson M, Nilsson M, Uustal E, Lindberg I (2018). 'A worse nightmare than expected' - a Swedish qualitative study of women's experiences two months after obstetric anal sphincter muscle injury. Midwifery.

[26] United Nations. The Sustainable Development Goals Report 2022. New York City: United Nations; 2022. Cited 2024 Feb 24. Available from: https://unstats.un.org/sdgs/report/2022/.

[27] Fahy K, Foureur M, Hastie C (2008). Birth territory and midwifery guardianship : theory for practice, education, and research.

[28] Walsh D, Christianson M, Stewart M (2015). Why midwives should be feminists. Midirs Midwifery Digest.

[29] Buchanan K, Newnham E, Geraghty S, Whitehead L (2023). Navigating midwifery solidarity: a feminist participatory action research framework. Women Birth.

[30] Benoit B, Goldberg L, Campbell-Yeo M (2016). Infant feeding and maternal guilt: The application of a feminist phenomenological framework to guide clinician practices in breast feeding promotion. Midwifery.

[31] Westergren A, Edin K, Walsh D, Christianson M (2019). Autonomous and dependent-The dichotomy of birth: a feminist analysis of birth plans in Sweden. Midwifery.

[32] Alspaugh A, Barroso J, Reibel M, Phillips S (2020). Women's contraceptive perceptions, beliefs, and attitudes: an integrative review of qualitative research. J Midwifery Womens Health.

[33] Davis DL, Walker K (2010). Re-discovering the material body in midwifery through an exploration of theories of embodiment. Midwifery.

[34] Govender V, Penn-Kekana L (2008). Gender biases and discrimination: a review of health care interpersonal interactions. Global Public Health.

[35] Graneheim UH, Lundman B (2004). Qualitative content analysis in nursing research: concepts, procedures and measures to achieve trustworthiness. Nurse Educ Today..

[36] Graneheim UH, Lindgren BM, Lundman B (2017). Methodological challenges in qualitative content analysis: a discussion paper. Nurse Educ Today.

[37] Lindgren BM, Lundman B, Graneheim UH (2020). Abstraction and interpretation during the qualitative content analysis process. Int J Nurs Stud.

[38] Tong A, Sainsbury P, Craig J (2007). Consolidated criteria for reporting qualitative research (COREQ): a 32-item checklist for interviews and focus groups. Int J Qual Health Care.

[39] Ministry of Health and Social Affairs. Hälso- och sjukvårdslag (2017:30) [Health and Medical Care Act]. Stockholm: The Swedish Riksdag; 2017. Cited 2024 Maj 8. Available from: https://www.riksdagen.se/sv/dokument-och-lagar/dokument/svensk-forfattningssamling/halso-och-sjukvardslag-201730_sfs-2017-30/.

[40] The Swedish Social Insurance Agency. Parental benefits. 2023. Cited 2024 Feb 20. Available from: https://www.forsakringskassan.se/english/parents/when-the-child-is-born/parental-benefit.

[41] The Swedish Association of Midwives. Kompetensbeskrivning för legitimerad barnmorska [Description of competences for registered midwives]. Stockholm: The Swedish Association of Midwives; 2018. Updated 2019 Jan. Cited 2024 Feb 20. Available from: https://storage.googleapis.com/barnmorskeforbundet-se/uploads/2020/04/Kompetensbeskrivning-for-legitimerad-barnmorska.pdf.

[42] Bäckenbottenutbildning [Pelvic floor education]. Utbildningsmaterial: Uppföljning [Educational material: Follow-up]. 2022. Cited 2024 Maj 8. Available from: https://backenbottenutbildning.se/index.php/utbildningsmaterial/uppfoljning.

[43] GynOp [The Swedish National Quality Register of Gynecological Surgery]. About GynOp. 2023. Cited 2024 Feb 20. Available from: https://www.gynop.se/home/about-gynop/.

[44] Öhman H, Schesny S, Bearth L, Löwendahl J. Förlossningsskadad? Du är inte ensam! [Injured at childbirth? You are not alone!’]. 2014. Cited 2024 Feb 20. Available from: https://www.facebook.com/groups/forlossningsskadad/.

[45] Vanderbilt University. About REDCap. Nashville: Vanderbilt; 2023. Updated 2023. Cited 2024 Feb 20. Available from: https://projectredcap.org/about/.

[46] Kvale S, Brinkmann S (2009). Interviews: learning the craft of qualitative research interviewing.

[47] Keen S, Lomeli-Rodriguez M, Joffe H (2022). From challenge to opportunity: virtual qualitative research during COVID-19 and beyond. Int J Qual Methods.

[48] Thunberg S, Arnell L (2022). Pioneering the use of technologies in qualitative research - A research review of the use of digital interviews. Int J Soc Res Methodol.

[49] MAXQDA. Why MAXQDA? Berlin: VERBI Software GmbH; 2024. Cited 2024 Maj 8. Available from: https://www.maxqda.com/why-maxqda.

[50] Microsoft. Microsoft Excel. Redmond: Microsoft; 2024. Cited 2024 Maj 8. Available from: https://www.microsoft.com/sv-se/microsoft-365/excel?market=se.

[51] Griffin GA (2017). Dictionary of Gender Studies.

[52] Abhyankar P, Uny I, Semple K, Wane S, Hagen S, Wilkinson J (2019). Women's experiences of receiving care for pelvic organ prolapse: a qualitative study. BMC Womens Health.

[53] Scott KD, Hintz EA, Harris TM (2022). "Having pain is normal": How talk about chronic pelvic and genital pain reflects messages from menarche. Health Commun.

[54] Pettersson A, Bertero CM (2020). How women with endometriosis experience health care encounters. Womens Health Rep (New Rochelle).

[55] Heitmann K, Svendsen HC, Sporsheim IH, Holst L (2016). Nausea in pregnancy: attitudes among pregnant women and general practitioners on treatment and pregnancy care. Scand J Prim Health Care.

[56] Roper JC, Amber N, Wan OYK, Sultan AH, Thakar R (2020). Review of available national guidelines for obstetric anal sphincter injury. Int Urogynecol J.

[57] The Australian Commission on Safety and Quality in Health Care. Third and Fourth Degree Perineal Tears Clinical Care Standard. Sydney: The Australian Commission on Safety and Quality in Health Care; 2021. Cited 2024 Feb 20. Available from: https://www.safetyandquality.gov.au/standards/clinical-care-standards/third-and-fourth-degree-perineal-tears-clinical-care-standard.

[58] Foucault M, Sheridan A (2003). The birth of the clinic: an archaeology of medical perception.

[59] Scarf VL, Rossiter C, Vedam S, Dahlen HG, Ellwood D, Forster D (2018). Maternal and perinatal outcomes by planned place of birth among women with low-risk pregnancies in high-income countries: a systematic review and meta-analysis. Midwifery.

[60] Offerhaus P, Jans S, Hukkelhoven C, de Vries R, Nieuwenhuijze M (2020). Women's characteristics and care outcomes of caseload midwifery care in the Netherlands: a retrospective cohort study. BMC Pregnancy Childbirth.

[61] Forster DA, McLachlan HL, Davey MA, Biro MA, Farrell T, Gold L (2016). Continuity of care by a primary midwife (caseload midwifery) increases women's satisfaction with antenatal, intrapartum and postpartum care: results from the COSMOS randomised controlled trial. BMC Pregnancy Childbirth.

[62] Brady S, Lee N, Gibbons K, Bogossian F (2019). Woman-centred care: an integrative review of the empirical literature. Int J Nurs Stud.

[63] Findlay D (1992). The medical gaze: medical models, power, and women's health. Atlantis.

[64] Samulowitz A, Gremyr I, Eriksson E, Hensing G (2018). "Brave Men" and "Emotional Women": a theory-guided literature review on gender bias in health care and gendered norms towards patients with chronic pain. Pain Res Manag.

[65] Fielding-Singh P, Dmowska A. Obstetric gaslighting and the denial of mothers’ realities. Soc Sci Med (1982). 2022;301:114938. 10.1016/j.socscimed.2022.114938 PMC9167791 35395611

[66] Sebring JCH (2021). Towards a sociological understanding of medical gaslighting in western health care. Sociol Health Ill.

[67] Jones CE (2015). Wandering wombs and "female troubles": the hysterical origins, symptoms, and treatments of endometriosis. Women Stud.

[68] Tasca C, Rapetti M, Carta MG, Fadda B (2012). Women and hysteria in the history of mental health. Clin Pract Epidemiol Ment Health.

[69] Young K, Fisher J, Kirkman M (2019). "Do mad people get endo or does endo make you mad?": Clinicians' discursive constructions of Medicine and women with endometriosis. Fem Psychol.

[70] The Swedish Ministry of Health and Social Affairs. Överenskommelse om en förbättrad förlossningsvård och insatser för kvinnors hälsa [Agreement on improving maternity care and women's health] [Internet]. Stockholm: The Swedish Government; 2015. S2015/07777/FS. [cited 2024 Maj 8]. Available from: https://www.regeringen.se/overenskommelser-och-avtal/2015/12/overenskommelse-om-en-forbattrad-forlossningsvard-och-insatser-for-kvinnors-halsa/.

[71] Boddy CR (2016). Sample size for qualitative research. Qual Mark Res.

[72] Malterud K, Siersma VD, Guassora AD (2016). Sample size in qualitative interview studies: guided by information power. Qual Health Res.

