# Supplementary material for: Overlooked by the obstetric gaze – how women with persistent health problems due to severe perineal trauma experience encounters with healthcare services: a qualitative study
Source: BMC Health Serv Res. 2024 May 9;24:610. doi: 10.1186/s12913-024-11037-5 (PMC11084138; doi:10.1186/s12913-024-11037-5)
Supplement: Supplementary file 1 — Additional file 1. Semi-structured interview guide for individual interviews; contains interview questions aimed at highlighting the experience of everyday life and working life after suffering 3rd or 4th degree perineal laceration at childbirth (i.e., severe perineal trauma [SPT]). [file 12913_2024_11037_MOESM1_ESM.pdf]

Additional file 1

Semi-structured interview guide for individual interviews

Everyday life

Interview questions aimed at highlighting the experience of everyday life after suffering 3rd or 4th degree perineal laceration at childbirth (i.e., severe perineal trauma [SPT]).

Broad, open-ended introductory question

Tell me what it is like to live with health problems due to SPT.

Supplementary questions

In what ways have health problems due to SPT affected your everyday life?

In what ways have health problems due to SPT affected your daily physical activities?

In what ways have you adapted your everyday life due to health problems related to SPT?

How do health problems due to SPT affect your relationship with your partner?

How do health problems due to SPT affect your relationship with your immediate family?

How have health problems due to SPT affected your social life (with friends or acquaintances)?

Describe how people around you have reacted to your health problems due to SPT.

How do you feel about health problems due to SPT impacting on your everyday life?

How do you envision your future?

Is there anything else you would like to tell me about your experiences of living with health problems due to SPT and if so, what?

Working life

Interview questions aimed at highlighting the experiences of working life after suffering 3rd or 4th degree perineal laceration at childbirth (i.e., severe perineal trauma [SPT]).

Broad, open-ended introductory question

Tell me how health problems due to SPT have affected your work.

Supplementary questions

In what ways have health problems due to SPT affected you physically in your work?

In what ways have health problems due to SPT affected you emotionally in your work?

In what ways have you adapted your working life due to health problems related to SPT?

Question for women with experiences of sick leave: what circumstances do you feel led to your sick leave?

Do you think that you should have been on sick leave because of your health problems due to SPT?

What have you chosen to do in terms of telling/not telling your manager and colleagues about your problems?

What support have you received from your colleagues or your manager?

What support would you have liked to have received at your workplace?

How has your ability to work affected other parts of your life?

How do you feel about the impact of your condition on your working life?

How do you envision the future in terms of your work and your ability to work?

Is there anything else you want to tell me about your perception of your work ability, and if so, what?

Follow-up questions

Could you please describe your experience in more detail?

Please tell me more about this experience. What were you thinking at the time? How did you feel?

Give some examples of your experience in this context.

What do you think could be the reason why you feel this way? How do you feel about... when you talk about this?

Describe how your experiences have changed over time/longer term.
